# Defining Bayley Scales of Infant and Toddler Development Third Edition (BSITD-III) meaningful change and item relevance in children with neuronopathic MPS II: a caregiver interview-based study

**DOI:** 10.1186/s13023-026-04205-9

**Published:** 2026-01-21

**Authors:** Dawn Phillips, Catherine Wilson, Vivian Fernandez

**Affiliations:** REGENXBIO Inc., 9804 Medical Center Drive, Rockville, MD 20850 USA

**Keywords:** MPS II, Neuronopathic, Bayley, BSITD-III, Developmental function, Caregivers, Meaningful change

## Abstract

**Background:**

Neuronopathic mucopolysaccharidosis type II (MPS II) is a rare genetic disorder characterized by multisystem impairments that include profound cognitive and behavioral impairment and global developmental delay. Developmental function is considered a sensitive indicator of disease progression and a relevant outcome to evaluate the efficacy of existing and novel therapies. The Bayley Scales of Infant and Toddler Development, Third Edition (BSITD-III) has been recommended by expert consensus to assess treatment effect in the disease. The current study aimed to better understand what constitutes a meaningful change on the BSITD-III in response to treatment of neuronopathic MPS II according to caregivers and to investigate the functional relevance of items on the BSITD-III to daily function. Ten semi-structured interviews were conducted with caregivers of children aged 2–6 years old in the UK and USA. The interviews included hypothetical vignettes describing a change on the BSITD-III in response to an intervention over 1 year. Caregivers were asked to rate the level of change, discuss whether the changes were clinically meaningful and to describe any relevance to daily function. Qualitative and quantitative analyses were undertaken.

**Results:**

Interviews involved 12 caregivers of 11 children. Caregiver perceptions support a meaningful change on the BSITD-III being as small as a change of 1–2 months in Age Equivalent values: increases in age-equivalent scores of 2 months were reported to be associated with meaningful changes (Caregiver Global Impression of Change rating of minimal or greater improvement) over 1 year by 97.1% of caregivers (all subtests) and increases of 1 month for the Fine Motor and Expressive Communication subtests were considered meaningful by 81.8% and 90.9% of caregivers, respectively. Caregivers reported that the BSITD-III Cognitive, Expressive Communication, and Fine Motor items outlined in the hypothetical vignettes were functionally relevant to their children with MPS II and provided practical examples of item relevance in their child’s daily activities.

**Conclusions:**

These findings support the use of BSITD-III as an appropriate clinical outcome assessment for measuring changes in developmental skills in children with neuronopathic MPS II.

## Background

Mucopolysaccharidoses type II (MPS II, Hunter syndrome; OMIM #309900) is a rare and progressive lysosomal storage disorder caused by the deficient activity of the enzyme iduronate 2-sulfatase (I2S) due to X-linked recessive mutations in the *IDS* gene [1]. The more common and severe form is neuronopathic MPS II, which is characterized by neurological involvement [2, 3], leading to progressive cognitive and behavioral issues and global developmental delay with hyperactive behavior being highly prevalent [2, 4, 5].

Although there is a spectrum of disease severity and a variable rate of progression, neurodevelopmental function follows the course of a delay phase, when children are still acquiring skills but at a slower rate than their peers and deviation increases from normal function, a plateau phase at an average age of 4 to 4.5 years, and then a progressive neurodevelopmental decline [6, 7]. Phillips et al. [8] reported that development may appear normal for the first few months but that natural history data indicate that skill acquisition may slow from as early as 6–9 months of age. The study showed that mean developmental score trajectories for global domains (expressive language, receptive language, fine motor, and visual reception) were one standard deviation below the normative mean at chronological ages of 12.8–19.5 months and two standard deviations below at 24.0–29.3 months [8].

The significant developmental decline in children with neuronopathic disease can be distressing and burdensome for families [5]. Caregivers participating in a U.S. Food and Drug Administration (FDA) MPS II listening session on February 2020, identified cognitive delay, communication, and adaptive skills as the most burdensome symptoms [9].

Developmental function is considered a sensitive indicator of disease progression in MPS II and a relevant outcome to evaluate the efficacy of therapies for this disorder [10–12]. It is therefore imperative that instruments used to assess developmental function are relevant to MPS II and able to reflect meaningful changes. Experts have reached consensus for the use of the Bayley Scales of Infant and Toddler Development, Third Edition (BSITD-III [13]) to assess the effect of treatment on developmental outcomes in MPS I, II, and III [11, 12].

The BSITD-III is an instrument used to assess global developmental function of infants and young children aged 1–42 months [13]. A child with MPS II with a chronological age of ≥ 42 months but a developmental function of < 42 months may also be evaluated using the BSITD-III. The BSITD-III uses a series of play tasks with the purpose of identifying children with developmental delay in Cognitive, Receptive and Expressive Communication, and Gross and Fine Motor subtests. Assessments start with simple item tasks which increase in difficulty until they are beyond the child’s ability [14]. Table 1 outlines the range of scores available and application to MPS II.

**Table 1:** BSITD-III scores and application to neuronopathic MPS II

BSITD-III Scores		
Description [13]	Limitations/Challenges [6, 24]	Application to Neuronopathic MPS II
**Raw**		
Represents the total number of items credited for each subtest	Raw scores do not represent an equal interval scale. Each subtest has a different number of total items; Cognitive (91 items), Receptive Communication (49 items), Expressive Communication (48 items), Fine Motor (66 items), Gross Motor (72 items) Difficult to compare raw score function between BSITD-III subtests or to compare to other developmental assessments	Illustrate skill acquisition
**Scale**		
Derived from the raw scores and range from 1–19 with a mean of 10 and a SD of 3 Calculated for each subtest Describe function relative to a normative reference sample that is available until a chronological age of 42 months Represent the rate of skill acquisition	Use is not applicable to children with advanced disease stage. Irreversible central nervous changes impact the rate of skill acquisition in response to treatment intervention. If skill acquisition is present but slower than the children in the normative sample, scale scores will decline, and alternative values are required to interpret change	Used to describe the distribution of the sample and to stratify participants at baseline Useful for defining a positive efficacy response in patients ≤ 42 months old with developmental function at or above − 2SD from the normative mean. Maintenance of function within 2SD from the normative requires a rate of skill acquisition similar to typically developing children in the normative sample An increase in scale from baseline is not necessary to define a positive efficacy response. Stability or even a small decline in scale scores is representative of a rate of skill acquisition similar to typically developing same age peers
**Composite Scores**		
Derived from the sum of subtest scale scores Generated for the Language (Expressive and Receptive Communication subtests), Motor (Fine and Gross Motor subtests), and Cognitive subtest Scaled to a metric with a mean of 100 and a SD of 15	Reference a normative sample and have the same limitations as scale scores in children with developmental function below − 2SD from the normative mean Composite scores for language and motor include more than one subtest and use restricts ability to determine which subtest is driving a change in scores	Use is most relevant in children with MPS II with developmental function at or above − 2SD from the normative mean
**Developmental Age Equivalents (AEq)**		
Average age in months in which a given total raw score is typical Available for Cognitive, Receptive and Expressive Communication, Fine and Gross Motor subtests	AEq scores are not an equal interval measure	Used to interpret skill acquisition across subtests Use in MPS II is most applicable to patients older than 42 months or with function below − 2SD from the normative mean
**Growth Scale Values (GSV)**		
Values are based on the subtest total raw score GSVs range from 200–800 Used to plot the child’s growth over time for each subtest Represent an equal interval scale	As an equal interval scale, the amount of change can be compared between subtests but actual GSV values between subtests cannot be compared because each subtest has different totals Used to illustrate skill acquisition but additional values are required to make a comparison to natural history data. In the natural history, children in the delay phase will still be acquiring skills even though the rate of skill acquisition is much slower than typically developing children. The GSV will increase but it cannot be determined if the magnitude of change exceeds the natural history	Use is most applicable in MPS II with patients with baseline function below − 2SD

AEq: age equivalents; BSITD-III: Bayley Scales of Infant and Toddler Development; GSV: growth scale values; MPS: mucopolysaccharidosis; SD: standard deviation

The aims of the current study were to better understand, from the caregiver perspective, what might constitute a meaningful change in response to treatment in patients with neuronopathic MPS II and to explore the functional relevance of items on the BSITD-III to the daily lives of these patients and their caregivers.

## Methods

### Recruitment

Caregivers in the USA were recruited by the USA National MPS Society and Project Alive, and in the UK by Rare Disease Research Partners (RDRP) in collaboration with the UK MPS Society. Caregivers who met the inclusion criteria were invited to participate via phone and email. To be eligible, caregivers had to be aged ≥ 18 years, care for a child/ children aged 2–6 years old with neuronopathic MPS II, speak English, and be willing and able to provide informed consent to participate. Only caregivers of patients not involved in a sponsored investigational intervention study were eligible. Eligible caregivers were provided with a participation information sheet before the interview and gave informed consent to participate, for interviews to be audio recorded, for quotes to be used verbatim and, in the UK only, for contact details to be shared with the study sponsor to undertake the interviews.

### Study design

A semi-structured interview guide was developed that included hypothetical vignettes outlining a change in response to intervention over 1 year on the BSITD-III. Interviews were conducted face to face or via video conference between August and November 2022. The interviews were audio recorded and transcribed verbatim for analysis.

During the interviews, caregivers were shown four vignettes describing hypothetical examples of a child’s function on the Cognitive, Expressive Communication, and Fine Motor subtests of the BSITD-III at baseline and 1 year after a hypothetical treatment intervention. For each vignette, the chronological age, raw, and age equivalent scores (AEq) were provided at baseline and compared to the same values 1 year post-treatment. The vignettes were created to represent different functional levels and were based on a clinical sample of patients (Table 2). Caregivers were asked to rate the level of change using the Caregiver Global Impression of Change (CaGI-C [15]) and were provided with descriptions of each level of change (Table 3). Caregivers were also asked to discuss if they considered the developmental changes clinically meaningful and what the functional impacts of these skills may be in daily life.

**Table 2:** Hypothetical vignettes of the BSITD-III discussed during caregiver interviews

	Baseline	1-year post treatment
**VIGNETTE 1**		
Chronological age	52 m	64 m
**Cognitive domain**		
Raw score	43	47
AEq	13 m	15 m
Developmental skills	• Puts one block in cup • Dumps blocks out of cup • Holds two blocks in hand and attempts third • Pushes toy car • Suspends ring on string • Removes pellets from bottle • Squeezes toy to make sound	Credit for additional items, including: • Finds hidden toy under washcloth • Retrieves object through open end of clear box
**Expressive Communication domain**		
Raw score	13	16
AEq	11 m	13 m
Developmental skills	• Gets direction of caregiver, directs attention and points to object • Uses gestures • Jabbers, uses one-word approximations • Has one vowel consonant blend	Credit for additional items, including: • Participates in a play routine • Imitates word • Uses two words appropriately Items not credited: • Directs attention of other person
**Fine Motor domain**		
Raw score	34	35
AEq	18 m	20 m
Developmental skills	• Holds crayon and makes random stroke on paper • Stacks two blocks • Puts ten pellets into bottle	Credit for additional items, including: • Pulls connecting blocks apart
**VIGNETTE 2**		
Chronological age	24 m	36 m
**Cognitive domain**		
Raw score	56	64
AEq	20 m	25 m
Developmental skills	• Places three puzzle pieces • Finds a hidden object under a washcloth when displaced • Put nine blocks in a cup and six pegs in the pegboard • Listens to a short story	Credit for additional items, including: • Puts four smaller puzzle pieces into puzzle board • Puts puzzle pieces in board when the board is rotated • Constructs a three-piece puzzle without a board • Identifies matching pictures for an airplane, bike and tree • Plays pretend with objects. Uses washcloth as blanket for baby doll
**Expressive Communication domain**		
Raw score	22	30
AEq	18 m	24 m
Developmental skills	• Initiates play interaction • Says two words • Uses words to make needs known • Labels ball correctly • Combines word with gesture • Answers yes and no to questions	Credit for additional items, including: • Says eight words • Uses two-word utterances and occasionally multi-word utterances • Says “me” • Labels five pictures and three objects correctly
**Fine Motor domain**		
Raw score	36	41
AEq	21 m	26 m
Developmental skills	• Stacks three blocks • Completes random scribbling on paper and uses other hand to hold paper • Puts tiny pellets into bottle • Puts three coins into piggy bank	Credit for additional items, including: • Pulls connecting blocks apart • Improves grasp on crayon and draws a vertical and horizontal line and a circle • Stacks six blocks
**VIGNETTE 3**		
Chronological age	38 m	50 m
**Cognitive domain**		
Raw score	64	70
AEq	25 m	29 m
Developmental skills	• Puts four puzzles pieces into a puzzle board • Inserts three puzzle pieces when board is rotated • Puts six pegs into pegboard • Matches similar pictures • Listens to a short story • Imitates a two-step action • Matches three colors	Credit for additional items, including: • Assembles small puzzle without board • Plays pretend. Pretends block is an apple and feeds baby • Completes nine pieces on a board puzzle • Imitates a two-step task • Understands concept of one • Passes one block on request
**Expressive Communication domain**		
Raw score	26	30
AEq	21 m	24 m
Developmental skills	• Names one object and five pictures • Combines word with gesture • Answers yes and no • Imitates two-word utterance • Says “me”	Credit for additional items, including: • Says eight words • Says two-word utterance • Labels three objects correctly • Names one action picture in book
**Fine Motor domain**		
Raw score	38	41
AEq	23 m	27 m
Developmental skills	• Puts three coins in piggy bank • Pulls connecting blocks apart • Scribbles on paper and holds paper with other hand	Credit for additional items, including: • Stacks six blocks • Draws vertical and horizontal lines
**VIGNETTE 4**		
Chronological age	48 m	60 m
**Cognitive domain**		
Raw score	52	51
AEq	18 m	17 m
Developmental skills	• Puts one peg in pegboard • Plays with toys and pretends to drink from cup and eat from spoon • Puts one large circle in puzzle board • Puts one small circle in puzzle board • Finds object hidden under washcloth on the left and right	Loss of credit for one item: • Unable to put any small puzzle pieces into puzzle board
**Expressive Communication domain**		
Raw score	15	16
AEq	12 m	13 m
Developmental skills	• Participates in play routine • Has one consonant vowel blend • Jabbers • Uses one word • Directs attention of another	Credit for additional item: • Uses word to make wants known
**Fine Motor domain**		
Raw score	25	36
AEq	20 m	21 m
Developmental skills	• Holds crayon using thumb and fingers and imitates a stroke • Stacks two blocks • Puts ten tiny pellets into bottle • Pulls connecting blocks apart	Credit for additional item: • Stacks six blocks

AEq: age equivalent score; m = months

### Analysis

Quantitative and qualitative analyses of the interview transcripts were conducted by RDRP. Respondents were assigned a unique ID number and responses were aggregated and remained de-identified. Quantitative frequency analysis of change ratings chosen for each domain/subtest of each vignette was conducted. The degree of change in AEq for each domain/subtest of each vignette was then compared to the percentage of caregivers who felt the changes were meaningful. A meaningful change was defined as a CaGI-C rating of ‘minimally improved’, ‘much improved’, and ‘very much improved’. Change was also described by where on daily life the change was considered by caregivers to have an important impact (Table 3).

Qualitative responses were organized by caregivers’ choice of change rating per vignette and were analyzed using reflexive thematic analysis, in accordance with the six phases outlined by Braun and Clarke [16]. Analysis was conducted using an inductive approach using NVivo software. Open semantic and latent coding was undertaken collaboratively by two researchers to capture both explicit and underpinning meanings from the data. Initial codes were reviewed by the researchers and organized into themes which captured significant concepts and ideas, followed by an iterative process of defining and naming themes.

**Table 3:** Caregiver global impression of change (CaGI-C [15]*)

Level of change	Description
Very much improved	Large amount of improvement in either their symptoms, physical ability, ability to perform daily activities, social life, emotions and mental wellbeing, or overall health, which has had a large and important impact on their life.
Much improved	Medium to large amount of improvement, which has had a moderate and important impact on their life.
Minimally improved	Small amount of improvement, which has had a small and important impact on their life
No change	Not experienced any noticeable change
Minimally worse	Small amount of worsening which has had a small and important impact on their life.
Much worse	Medium to large amount of worsening, which has had a moderate and important impact on their life.
Very much worse	Very large amount of worsening, which has had a large and important impact on their life.

^*^For this study, a meaningful change was defined as a CaGI-C rating of ‘minimally improved’, ‘much improved’, and ‘very much improved

### Ethical considerations

This research was conducted in accordance with the British Healthcare Business Intelligence Association’s Legal & Ethical Guidelines for Market Research [17]. In line with these guidelines, this market research did not require approval by a Research Ethics Committee. The study was non-interventional, involving individuals recruited via patient organizations. All participants provided informed consent. The data collected was pseudonymized and participants were free to stop the interview at any point. Participants could indicate any shared content that they did not want cited within study summaries or manuscripts.

## Results

### Participant characteristics

Ten interviews (six in the USA and four in the UK) were conducted involving 12 caregivers of 11 children with neuronopathic MPS II. One respondent had two children with MPS II. For two children, both parents jointly took part in the interviews although in one case, one caregiver left the interview due to caring responsibilities (*n* = 12 is reported when this caregiver’s responses are included). All 11 children were male with ages ranging from 3 to 6 years old (age 3: *n* = 4; age 4: *n* = 4; age 5: *n* = 2; age 6: *n* = 1). No caregivers of 2-year-old children were recruited. The functional level of patients varied reflecting the heterogeneity of disease progression in 3–6-year-old children with neuronopathic MPS II.

### Caregivers’ perception of a meaningful change on the BSITD-III

#### Changes in AEq indicative of a meaningful change

Caregivers felt the AEqs of the children in the vignettes were a tangible and meaningful measure to understand the developmental changes that had taken place in 1 year compared to normal development and were relevant to the daily life and health experience of children with MPS II, although for some respondents, the perception of skill loss depended on the child’s chronological age. Table 4 provides a summary of caregiver ratings of meaningful changes on the hypothetical vignettes of the BSITD-III.

**Table 4:** Caregiver ratings of meaningful improvements on the BSITD-III domains. Change in AEq shows the difference in AEq from baseline to 1-year post treatment on the hypothetical vignettes (see Table 2)

Vignette number	Domain	Change in AEq (months, m)	Meaningful improvement* (% caregivers)
V1	Cognitive	2 m increase	97.1% (11/12)
	Expressive Communication	2 m increase	97.1% (11/12)
	Fine Motor	2 m increase	97.1% (11/12)
V2	Cognitive	5 m increase	100% (11/11)
	Expressive Communication	6 m increase	100% (11/11)
	Fine Motor	5 m increase	100% (11/11)
V3	Cognitive	4 m increase	100% (11/11)
	Expressive Communication	3 m increase	100% (11/11)
	Fine Motor	4 m increase	100% (11/11)
V4	Cognitive	1 m decrease	0% (0/11)
	Expressive Communication	1 m increase	90.9% (10/11)
	Fine Motor	1 m increase	81.8% (9/11)

*CaGI-C rating of minimally improved or greater

AEq: age equivalent scores; CaGI-C: Caregiver Global Impression of Change; m = months

#### Retention of existing skills

Caregivers agreed that the retention of existing skills were as important as gaining additional skills because it indicated that the child’s condition had not started to regress.

As a Hunter syndrome mother, I think that we have a different outlook on that and being able to just keep the skills that we have matters just as much.

Any cognitive gain is a gain […] in the Hunter world

It was frequently remarked that any improvement in cognitive functioning would be meaningful, given the progressive and irreversible nature of neuronopathic MPS II. Caregivers described that even minimal changes would provide reassurance of the child’s development and support lack of disease progression. An improvement was not only considered relative to physical performance. It was frequently mentioned that any type of gained skills represented a general improvement in the child’s attention span, ability to focus on tasks, and willingness to participate more actively in the world around them.

### Relevance of changes in the BSITD-III in the child’s daily life

During the interviews, caregivers considered the overall changes seen in the hypothetical vignettes in relation to daily function for children with MPS II. Cognitive, Expressive Communication, and Fine Motor concepts considered meaningful to caregivers are discussed below.

#### Cognition

The understanding of abstract concepts, and specifically pretend play, were described by caregivers as *“huge”* and *“impressive”* because the activity represents function in typical children, demonstrating an ability to assimilate knowledge from previous experiences and to coordinate interpretations of the world to visualize something that is not tangible. The items that measure object permanence, such as ‘hiding toys under washcloths’, were linked by caregivers to searching for items that are not directly in view. Puzzle items were deemed meaningful by 100% of caregivers as they would support the development of problem-solving skills. Caregivers found improvements in tasks that paired object manipulation with problem-solving meaningful. Examples of items include being able to use trial and error to ‘retrieve objects from a clear box’, ‘dump blocks out of a cup’, and ‘remove pellets from a bottle’. ‘Putting puzzle pieces on a board that was rotated’ demonstrated application of a previous experience with skill advancement.

Skills that show the ability to understand multiple steps were found supportive of sequencing of movement and activities in daily life.

That’s a very good skill to have picked up, because they’re now recognizing the processes of what is happening and in day-to-day life. Attending nursery, getting up, having their breakfast, getting dressed, having their teeth brushed, getting to school, following the school’s routine throughout the day and then coming home. So, it’s understanding that process is going to be all linked back to being able to understand smaller tasks.

Caregivers found that BSITD-III items that measure memory link to ability to utilize knowledge from past experiences to overcome everyday challenges. Examples highlighted by caregivers included the ability to ‘construct a three-piece puzzle without the board’ and ‘find hidden toy under washcloth’. Improved memory has functional relevance for children with MPS II, particularly in a school or nursery setting:

[…] looking for things…, especially at nursery, they’ll be asked to tidy up at the end of their session, what they’re playing with, to actually understand that they’re looking for the toys and to pick them up and putting them away.

Caregivers reported that children can become distressed when they do not remember or understand their environment or routines and improvements in memory can reduce frustration and improve behavior adaptability to environmental changes.

#### Expressive communication

Caregivers found items that required the child to label objects and actions or utilize words appropriately were important to the child’s awareness and understanding of their environment and were considered a crucial step to future language acquisition. For example, items that require naming of an action in the response book represented a *“huge”* step in identification and understanding of operational relationships between objects and actions and in the development of a general awareness of how the world works. Caregivers felt these understandings would support the child’s ability to engage in everyday life, educational settings, and further learning.

It’s significant getting more words, putting them in a context. When they’re able to label objects correctly… That’s then leading to, that’s a plane, so if I know that object is a plane, I’m going to then learn the word plane and I might use the word in, ‘plane sky’.

Improvements in language skills were deemed relevant to further learning, encouraging interactions, and gaining knowledge from others. The child’s ‘participation in a play routine’, ‘imitating a word’, and ‘using two words appropriately’, provided opportunities to learn through play, which would consolidate an understanding of real life.

“[…] a play-routine could be including roleplay. So, that’s them having to think about what sounds a dog might make or a car might make and putting real life into these play scenarios.”

Caregivers noted that communication was a particular challenge for children with MPS II and explained how difficult it can be when their child is unable to communicate and express themselves. An ability to communicate wants and needs was the most meaningful aspect of Expressive Communication change. Regression in communication can be emotionally and logistically challenging, as it restricts the caregiver’s understanding of the child’s needs.

It can bring so much more to their life through communication, even if it’s just a couple of words.

Communication skills were related to improvements in behavior as the ability to express wants and needs reduces children’s frustration and challenging and disruptive behaviors as they provide the child with a sense of security that their needs would be met.

If they don’t have all the words then and they want something […] Or they feel something, they don’t know how to get it out. And so, if those words are the way they’re feeling sad or hungry or tired, then you know exactly what they want, so they don’t go into tantrums.

[…] being able to express so you feel safe and confident in your space.

Caregivers expressed a desire for their children to be able to communicate with, and be understood, by people other than their main caregiver. They noted that their child’s limited language skills, vocabulary, and slurred speech could hinder their education and socialization as only those who knew the child well could interpret what they were trying to communicate. Several items on the Expressive Communication subtest were deemed relevant, for example, the ‘use of two words appropriately’ and the ability to ‘name objects correctly’, indicated that the child had a practical knowledge and more available tools to communicate clearly and accurately, potentially improving the child’s understanding and wellbeing in educational settings as it would support engagement with their peers and teachers.

A progression from using gestures, jabbering, using one-word approximations and having a one vowel consonant blend to being able to ‘imitate’ and ‘use two words appropriately’ was seen as a *“big leap in their abilities”* which indicated the child had progressed from *“early communication”*. A caregiver noted that they would be extremely pleased if their child gained an additional two words in a year and explained that any improvements in language skills, no matter how small, would be hugely meaningful and important.

I feel like I place more emphasis on the expressive language than maybe I should, because that’s our huge thing with [Child’s name]. I mean, if he would imitate a word and use two words appropriately, I would be just ecstatic. […] I don’t know if I’m alone in this, the language and loss of language, I feel like that’s just a huge thing with the Hunter syndrome, just the not talking and the not being able to express himself with language. So even just the small improvement of the two words in a year, that would mean so much to me. Absolutely.

High value was placed on the length of utterances the child was able to use rather than the total vocabulary as the use of short sentences would allow wider and more accurate communication and would represent a key milestone in language development. This skill represented a *“very big improvement”* that was relevant to everyday life as it indicated the ability to construct short sentences to express an idea or communicate their wants and needs more clearly to others, as a single word or verb could be misconstrued. This skill was also related to more positive interactions with peers *“in nursery or being out*,* […] the more words you’ve got*,* the better to actually be able to have a conversation with another child. Maybe be able to ask them to play.”*

#### Fine motor

Block stacking was meaningful to caregivers because it requires some understanding of the mechanical principles and forces involved in balancing that could be applied to other domains and settings and indicated spatial awareness, which *“is important for functionality in terms of play and social expression”*. They also thought that items such as block stacking involve a process with a consequence, were a tangible measure of coordination and translate into skills relevant to daily life.

He was only stacking two, and then probably thinking, don’t want to do too many more, they might fall over. He’s now thinking, I can do more, let’s see how many I can do before maybe they fall. So, he’s understanding that there’s ability to build up what he’s playing with and to see what the consequences are by stacking more.

Caregivers explained that children with MPS II are usually restricted in writing and drawing due to joint stiffness, carpal tunnel and cognitive ability. Improvements in functional skills relevant to learning to write, dexterity, control, and cognition were highly valued. For example, the strength of grip and control required to ‘pull a small block apart’ could be applied to holding and controlling a writing implement. The item ‘draws vertical and horizontal lines’ was seen as a precursor to writing as it indicated a development of skills from simple scribbling to being purposeful and coordinated. Caregivers felt that an ability to draw and write would be enriching as it would allow better participation in educational settings and foster creativity.

### Participation and socialization

Caregivers remarked that behavioral abnormalities associated with the cognitive decline seen in MPS II mean that children can be internally focused and reluctant or unable to engage in daily activities. Caregivers therefore considered that their child’s willingness to engage with tasks in a social setting was meaningful.

A number of items (‘passing a block on request’, ‘naming pictures’, ‘drawing vertical and horizontal lines’, ‘imitating a two-step task’) suggested improvements in the child’s ability to comprehend and follow directions which was considered to be an important skill which caregivers noted could be challenging for their own children and raised a number of safety concerns, particularly when outside of the home environment.

Being able to pass a block on request [is meaningful], being able to do what’s requested of them outside of school or even at home when somebody requests them to do something and being able to follow through on that, getting directions. Even safety issues outside, like, if they’re at a park being able to do something on request is huge.

Several items represented improvements in emotional intelligence that would allow children to engage with their unaffected peers in educational environments. For example, ‘pretending to use a washcloth as a blanket for a baby doll’ involved an *“emotional aspect of caregiving”* suggesting the child was able to understand and consider the emotional needs of others. This was particularly meaningful to parents, who often referenced a stronger desire for their child’s social development over their academic progress.

A desire to participate and communicate was referenced as a key component captured by the changes in several vignettes, particularly in the Expressive Communication domain, and was often more meaningful than technical improvements in language. Attempts to communicate, even if unintelligible, showed that the child had a desire to engage socially and participate in their world. For example, the item ‘participates in a play routine’ suggested the child’s confidence and willingness to socialize had improved, while ‘Says ‘me’’ showed the child had developed a better sense of *“their place in the world”* and their social role within school and their family.

### Independence

Issues with joint mobility and range of movement, particularly in the wrists and fingers, can make it difficult for children to independently complete daily activities such as getting dressed. Improved Fine Motor skills were strongly related to a functional ability to be independent in daily activities, reduction of caregiving burden and improvement in overall wellbeing for the child and their caregiver.

Items which indicated improvement in the child’s arm and hand range of motion and or dexterity included ‘draws horizontal and vertical lines’ and ‘improves grasp on crayon’. It was noted that activities such as dressing and feeding require controlled and versatile hand movements and improvements can support more independence in daily activities.

Being able to hold onto the ball whilst you’re walking to the park. […] having that control when you’re playing with the ball in the park. Going shopping, if you’re helping with your parents, to be able to pick products up off the shelf and putting them into the trolley carefully, not throwing them.

The child’s ability to pull apart a small connecting block was considered functionally relevant to school, play and daily activities. The action of pulling involved different muscles that were not utilized in the baseline items, demonstrating that the child had developed strength and functionality in their muscles. The cognitive and physical ability to perform the action of pulling could translate to opening drawers or food packages, picking up pencils, putting on shoes, holding cups, donning or removing clothes, and manipulating clasps.

The item ‘stacks six blocks’ indicated improved hand-eye coordination and cognitive understanding of balancing. Parents equated improved hand-eye coordination to completion of daily tasks such as tidying up toys. Improved cognitive understanding paired with improved fine motor skills was relevant to level of independence, and specifically the level of trial and error often required to overcome obstacles and successfully perform daily activities.

## Discussion

The U.S. Food and Drug Administration industry guidelines highlight the need for clinical outcome assessments in clinical trials that are meaningful and relevant to patient’s lived experience of disease manifestations [18]. These caregiver interviews represent an effort to further understand the caregiver perspective on the clinical meaningfulness of the BSITD-III and the relevance of the items used in this assessment to daily activity. Caregivers supported the relevance of the BSITD-III Cognitive, Expressive Communication, and Fine Motor items presented in the hypothetical vignettes as representing a meaningful measure of a child’s development and linked the items to corresponding experiences of daily function in children with neuronopathic MPS II. The functional level of patients in the vignettes varied, reflecting the heterogeneity of disease progression in these children aged 3–6 years; therefore, our study showed the BSITD-III development concepts are relevant for children of this age group with neuronopathic MPS II and could potentially be applied more widely to other MPS disorders beyond the age of 3 years.

The caregiver perspectives presented in this manuscript are based on hypothetical vignettes. Longitudinal performance-based assessment with the BSITD within the context of a clinical trial would provide additional context on the relationship between the items, daily function and meaningful change. In conjunction with the BSITD, a comprehensive assessment of children with MPS II would also include a measure of adaptive behavior to further understand the role behavior, social awareness and peer interaction plays in daily function.

Neurodevelopmental decline and associated behavioral abnormalities, including autistic-like behaviors, loss of fear, impulsivity and hyperactivity, have a considerable impact on both the patient with MPS II and the family, significantly reducing their quality of life [2, 5, 19–23]. The BSITD-III has become the most widely used standardized tool for assessing neurodevelopment in early childhood [13, 14], and in patients with MPS II, has been used extensively and shown to be feasible and sensitive to changes, leading to its recommendation for assessing treatment effect [11, 12]. AEqs, available for the Cognitive, Receptive and Expressive Communication and Fine and Gross Motor subtests in BSITD-III, are generally considered the optimal measure for assessing the neurocognitive and adaptive growth of children particularly those with neuronopathic disease [6]. BSITD-III AEqs for the Cognitive subtest have also been shown to be highly correlated with parent-reported scores on the Vineland Adaptive Behavior Scales-II for patients with MPS disorders, further supporting their validity [24].

Our results add to this body of evidence supporting the use of the BSITD-III by determining a threshold for decline or improvement in the BSITD-III AEqs for patients with MPS II and showing that a small change in AEq of 2 months (or above) was meaningful to caregivers. Additionally, the loss of a skill was not as meaningful to caregivers if other skills were maintained or gained. These findings show that the BSITD-III AEqs were sufficiently sensitive to reflect changes that were meaningful to caregivers and highlight the need to take into consideration small changes which may be important for patients and their families. As noted by the caregivers, the fact that even small changes in BSITD-II AEq scores may represent the acquisition of developmental skills that are impactful reflects the severity and expected progression of disease, across multiple neurodevelopmental domains, in these young children with neuronopathic MPS II [8, 22]. A holistic approach may be needed when determining treatment outcomes which accounts for this effect that a small gain in skills has on the physical, psychological, social, and spiritual wellbeing of patients and their caregivers [25].

Caregiver perceptions of the level of changes in response to intervention were assessed with the CaGI-C, a widely used tool that allows rating of the changes on a seven-category scale ranging from ‘very much worse’ to ‘very much improved’ [15, 26]. CaGI-C is considered particularly valuable for young patients with rare diseases that are heterogeneous, affect multiple organ systems and have complex symptomology [26]. To further increase capture of all aspects of the experience of caregivers, open-ended, exploratory questions were also asked to determine whether caregivers considered the developmental changes clinically meaningful and what the functional impacts of these skills were on daily activities. Overall, caregivers reported that the BSTID-III Cognitive, Expressive Communication and Fine Motor items outlined in the hypothetical vignettes were functionally relevant to their child with MPS II, supporting the use of this tool for capturing meaningful changes that are relevant to everyday experiences and the quality of life of children with MPS II and their caregivers.

On considering the relevance of BSITD-III to daily life, caregivers described the importance and positive impact of their child becoming able to solve puzzles/problems, follow multiple steps/sequences and use knowledge from past experiences as these skills are all relevant to being able to participate in daily activities and overcome everyday challenges; being able to understand and participate in daily routines then also reduces frustration and improves behavior, with associated positive impacts for both the children and their families. Similarly, in the Expressive Communication subtest, caregivers felt that the ability to label objects/actions or use words appropriately suggested an understanding that would increase engagement with everyday life. The beneficial impacts of improvements in understanding and participation in the world among patients with MPS II were also noted in a recent caregiver exit interview following a clinical trial [27].

Previous studies have reported the limitations, challenges and burden of communication and language problems in children with MPS disorders [27, 28]. Caregivers in our study considered increases in communication skills to be associated with improvements in behavior and emphasized a strong desire for their child to be able to express themselves, with even small changes in language being reported to have a considerable impact. There is a recognized burden of social isolation and loss of independence on both patients with MPS disorders and their caregivers [20, 28] hence Fine Motor skills, such as block stacking, were particularly meaningful to caregivers as they indicated a functionality that may have implications for completion of daily tasks and increased independence, as well as potentially increased participation in educational settings and in play, and therefore socialization. The ability to perform tasks that demonstrated a willingness to engage were also considered meaningful because of the implication for improved socialization and interaction with peers.

A limitation of this research study is that a lack of saturation was found during analysis of the interviews, but this is expected in studies involving patients with rare diseases as recruitment is limited by the small sample size of the population. In addition, it was challenging to recruit caregivers of patients with neuronopathic MPS II who were not currently participating in a clinical study. Although saturation was not achieved, the key themes were consistent across caregivers. Since MPS II is a very heterogeneous disease, caregiver perceptions may differ depending on previous experience of disease presentation.

## Conclusions

Caregiver perceptions support a meaningful change on the BSITD-III being as small as a change in AEq of 1–2 months. Caregivers found that the BSITD-III Cognitive, Expressive Communication, and Fine Motor items outlined in the hypothetical vignettes represented items that were functionally relevant to their children. The caregivers provided practical examples of item relevance within their child’s daily activities. This supports the BSITD-III being an appropriate clinical outcome assessment to measure developmental skills in children with neuronopathic MPS II.

## Data Availability

The datasets and interviews generated and/or analyzed during the current study are not publicly available due to patient confidentiality.
